# Correction: Li et al. TRIM10 Is Downregulated in Acute Myeloid Leukemia and Plays a Tumor Suppressive Role via Regulating NF-κB Pathway. *Cancers* 2023, *15*, 417

**DOI:** 10.3390/cancers16162824

**Published:** 2024-08-12

**Authors:** Lin Li, Qi Li, Zhengrong Zou, Zoufang Huang, Yijian Chen

**Affiliations:** 1 Suzhou Medical College of Soochow University, Suzhou 215123, China; 18979740225@163.com; 2 Department of Hematology, The First Affiliated Hospital of Gannan Medical University, Ganzhou 341000, China; nfyyjsjj@126.com; 3 Basic Medicine Department, Chuxiong Medical and Pharmaceutical College, Chuxiong 675005, China; 15559705116@163.com; 4 Department of Emergency, The First Affiliated Hospital of Gannan Medical University, Ganzhou 341000, China; zzrboy87@163.com

## Error in Figure

In the original publication [1], there was a mistake in Figure 6G, the CHI 1 μM group of K562 cells as published. Making a mistake of placing a duplicate flow chart of (Figure 3E, the Vector group of HL60 cells) in the place of (Figure 6G, the CHI 1 μM group of K562 cells). The corrected Figure 6G, the CHI 1 μM group of K562 cells appears below. The authors apologize for any inconvenience caused and state that the scientific conclusions are unaffected. This correction was approved by the Academic Editor. The original publication has also been updated.

## Figures and Tables

**Figure 6:**
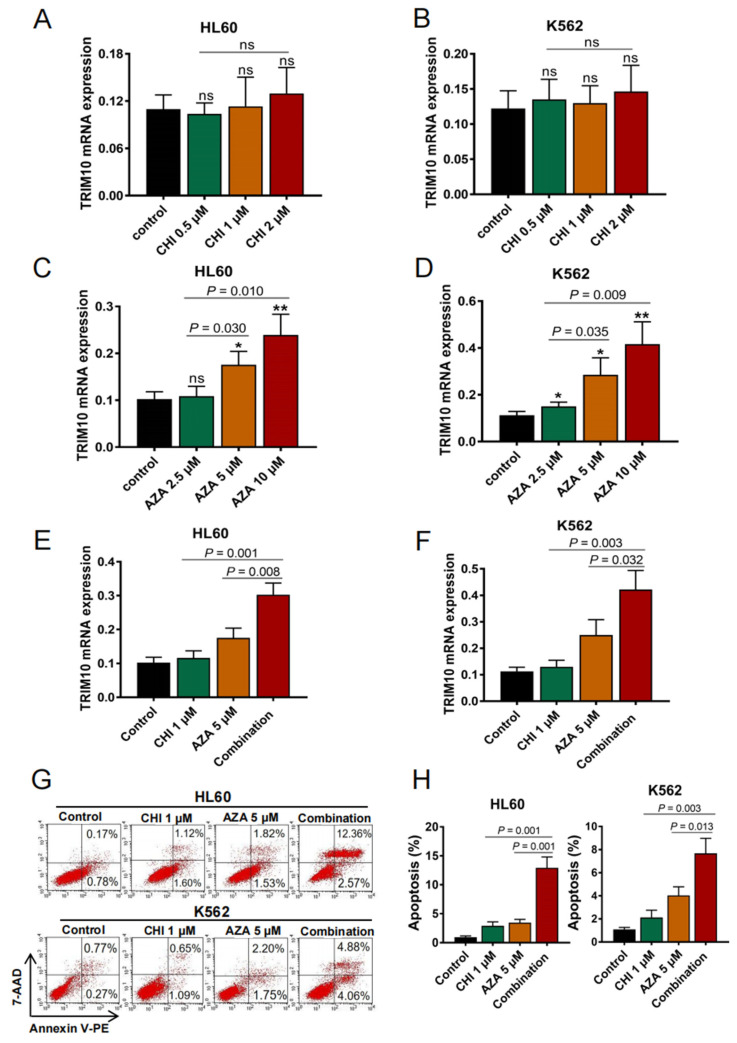
Combination of azacitidine (AZA) and chidamide (CHI) results in upregulation of TRIM10 expression and remarkable apoptosis in AML cells. (**A**,**B**) qRT-PCR analysis of TRIM10 expression after treatment with different dose of CHI for 48 h. (**C**,**D**) qRT-PCR analysis of TRIM10 expression after treatment with different dose of azacitidine for 48 h. (**E**,**F**) qRT-PCR analysis of TRIM10 expression after treatment with 5 μM azacitidine and 1 μM chidamide singly or in combination for 48 h. (**G**,**H**) Annexin V-PE/7-AAD double staining analysis of HL60 and K562 cells treated with 5 μM azacitidine and/or 1 μM chidamide for 48 h. Percentages of AML cell apoptosis based on three independent experiments. * *p* < 0.05; ** *p* < 0.01 (Student’s *t*-test).
